# Machine learning based algorithms to impute PaO_2_ from SpO_2_ values and development of an online calculator

**DOI:** 10.1038/s41598-022-12419-7

**Published:** 2022-05-17

**Authors:** Shuangxia Ren, Jill A. Zupetic, Mohammadreza Tabary, Rebecca DeSensi, Mehdi Nouraie, Xinghua Lu, Richard D. Boyce, Janet S. Lee

**Affiliations:** 1 Intelligent Systems Program, University of Pittsburgh, Pittsburgh, PA USA; 2 Division of Pulmonary, Allergy, and Critical Care Medicine, University of Pittsburgh, Pittsburgh, PA USA; 3 Department of Medicine, Acute Lung Injury Center of Excellence, University of Pittsburgh, NW628 Montefiore University Hospital, 3459 Fifth Avenue, Pittsburgh, PA 15213 USA; 4 Department of Biomedical Informatics, University of Pittsburgh, Pittsburgh, PA USA

**Keywords:** Machine learning, Respiratory tract diseases

## Abstract

We created an online calculator using machine learning (ML) algorithms to impute the partial pressure of oxygen (PaO_2_)/fraction of delivered oxygen (FiO_2_) ratio using the non-invasive peripheral saturation of oxygen (SpO_2_) and compared the accuracy of the ML models we developed to published equations. We generated three ML algorithms (neural network, regression, and kernel-based methods) using seven clinical variable features (N = 9900 ICU events) and subsequently three features (N = 20,198 ICU events) as input into the models. Data from mechanically ventilated ICU patients were obtained from the publicly available Medical Information Mart for Intensive Care (MIMIC III) database and used for analysis. Compared to seven features, three features (SpO_2_, FiO_2_ and PEEP) were sufficient to impute PaO_2_ from the SpO_2_. Any of the ML models enabled imputation of PaO_2_ from the SpO_2_ with lower error and showed greater accuracy in predicting PaO_2_/FiO_2_ ≤ 150 compared to the previously published log-linear and non-linear equations. To address potential hidden hypoxemia that occurs more frequently in Black patients, we conducted sensitivity analysis and show ML models outperformed published equations in both Black and White patients. Imputation using data from an independent validation cohort of ICU patients (N = 133) showed greater accuracy with ML models.

## Introduction

The ratio of the partial pressure of oxygen (PaO_2_) to the fraction of oxygen (FiO_2_) delivered, or the PaO_2_/FiO_2_, is the reference standard measurement for the assessment of low blood oxygen levels, or hypoxemia, in mechanically ventilated patients with respiratory failure. The PaO_2_/FiO_2_ ratio (PF ratio) has predictive value for mortality in patients with acute respiratory distress syndrome (ARDS)^1^ and is also part of a severity index scoring system called the Sequential Organ Failure Assessment (SOFA) score that is used to predict severity of illness in patients with critical illness^2–4^. Additionally, the PF ratio is relevant to clinical decision-making including the decision to initiate prone positioning in ARDS patients with PF ratios ≤ 150^5^. Currently, measurement of the PF ratio requires invasive arterial blood gas (ABG) sampling and does not provide a continuous measure of the patient’s oxygenation. Increasingly, non-invasive monitoring with pulse oximetry is utilized instead of ABGs^6,7^, particularly in low-resource settings where ABG monitoring may not be readily available. In contrast to invasive blood gas sampling, the SpO_2_ (peripheral saturation of oxygen)/FiO_2_ ratio can be calculated without blood collection, arterial puncture, or blood gas analyzers and may serve as a surrogate for the PaO_2_/FiO_2_ ratio. Notably several studies have evaluated the SF ratio in children where non-invasive measurements are increasingly favored^8–10^.

A few studies have examined non-linear imputation of PaO_2_/FiO_2_ from SpO_2_/FiO_2_ measurements recorded at the same time^11,12^. These studies have reported that the accuracy of non-linear imputation is superior to log-linear or linear imputation, especially for moderate to severe hypoxemic respiratory failure with ARDS where the PF ratio is < 200^11,13^. However, in patients with respiratory failure requiring mechanical ventilation, the optimal equation for imputation of PaO_2_/FIO_2_ from the SpO_2_/FIO_2_ remains unclear. An algorithm to accurately impute the PaO_2_ from the SpO_2_ in mechanically ventilated patients would be beneficial for predictive modeling and clinical research to facilitate recruitment of patients for clinical trials if an ABG is not available. Ideally, this approach would include only variables that contribute to the relationship between SpO_2_ and PaO_2_ but would not require the same invasive ABG measurement as the PaO_2_. From the clinical perspective, SF ratio can be utilized as a surrogate for PF ratio to diagnose ARDS or ALI with less invasive nature and comparable reliability^14^.

The objective of this study is to develop a calculator utilizing machine learning algorithms to impute PaO_2_ using non-invasive SpO_2_ measurements from mechanically ventilated patients in the Medical Information Mart for Intensive Care (MIMIC) III database^15^ and to compare the accuracy of the machine learning models to the previously published non-linear and log-linear equations^11,13^. In this study, three common machine learning approaches (neural network^16^, regression^17^, and kernel-based methods^18,19^) were tested for regression and classification tasks using data available in MIMIC III^20^ with 7 clinical variable features and a subsequent 3-feature model. We created models to perform a regression task to impute PaO_2_ from SpO_2_ values and a classification task to predict patients with moderate to severe hypoxemic respiratory failure based on a cut-off of a predicted PF ratio ≤ 150^11^. Our overall hypothesis is that a machine learning algorithm would perform better in predicting the PaO_2_ from SpO_2_ across the entire span of SpO_2_ values when compared to the previously published equations.

## Methods

The MIMIC-III database v1.4 (https://mimic.physionet.org) is an openly available dataset developed by the Massachusetts Institute of Technology Lab for Computational Physiology^15^. It contains de-identified health data associated with approximately 40,000 intensive care unit admissions for patients admitted to critical care units in the Beth Israel Deaconess Medical Center between 2001 and 2012. MIMIC-III is a relational database that contains information on demographics, vital signs, mechanical ventilation status, laboratory tests, medications, and mortality. We also utilized a validation cohort obtained from an existing database of de-identified clinical information from intensive care unit patients with *Pseudomonas aeruginosa* respiratory isolates from 2 hospitals within the University of Pittsburgh Medical Center (UPMC). This dataset similarly contains information of demographics, mechanical ventilation status, ventilator parameters and laboratory tests. Our study utilizing the MIMIC-III database was determined as exempt by the University of Pittsburgh Institutional Review Board (STUDY19100068). The University of Pittsburgh Institutional Review Board approved the *Pseudomonas aeruginosa* ICU respiratory isolates database as waiver of informed consent (STUDY21030010) and also approved the use of this database as an independent validation cohort (STUDY21090073). All methods were carried out in accordance with relevant guidelines and regulations.

### Data processing

For the MIMIC-III database, we identified unique ICU encounters (icustay_id) with mechanical ventilation status. We next identified the lab event PaO_2_ and chart event SpO_2_ occurring at the same time of the mechanical ventilation status. In order to minimize error between matched PaO_2_ and SpO_2_, we constrained the time gap between the lab event PaO_2_ and the chart event SpO_2_ to be no more than 30 min. To minimize repeated sampling from the same subjects, we restricted the search of PaO_2_ measurements to the first 24 h of mechanical ventilation and obtained the first PaO_2_ recorded within this time frame. For chart events including tidal volume (TV), positive end-expiratory pressure (PEEP), FiO_2_, temperature, and mean arterial pressure (MAP), we constrained the time gap to within 2 h of the selected SpO_2_ measurement. If a patient was treated with vasoactive infusions, it was recorded as a categorical variable. Data extraction and processing methods are available at https://github.com/renshuangxia/PaO2PredictorDjango^21^. The online calculator is available at https://dikb.org/pa02-predictor.

For the 3-feature model in the UPMC validation cohort, the database was queried for unique ICU patients requiring mechanical ventilation. The validation set cases include 133 discrete individuals with ABGs obtained within 30 min of an SpO_2_ reading similar to the constraints defined in the MIMICS III derivation cohort.

### Machine learning methods for regression task

For the regression task we implemented 3 different models—a neural network model, a linear regression model, and support vector regression (SVR), a type of kernel-based modeling. For each model, we applied a tenfold cross-validation^22^.

For the neural network model, we tested different network structures and various numbers of features to arrive at two models used for comparison with the linear and support vector regression models. One model used seven input features and three hidden layers (16, 8, 5 neurons for layers 1–3). The other model used only three input features and two hidden layers (6, 3 neurons for layers 1 and 2). Both final models used a tangent activation function for all layers except the output layer which used a linear function in both models. Also, both models were trained for 200 epochs with Adam optimizer using gradient descent. The learning rate was 0.001 and the batch sizes were 50 for both models.

For the linear regression model, the output variable can be computed by a linear combination of the input variables. We trained the linear regression equation by the Ordinary Least Squares approach. We used the linear_model.LinearRegression method from scikit-learn 0.22 (https://scikit-learn.org/stable/) with default hyperparameters for predicting PaO_2_ values.

For the SVR model, we tested multiple kernels including linear kernel, polynomial kernel, and radical basis function kernel (RBF). Based on the performance in the training data, the RBF kernel was selected.

### Machine learning methods for classification task

We utilized PaO_2_/FiO_2_ ≤ 150, an accepted threshold previously utilized to capture patients with moderate to severe disease meeting the criteria for ARDS^11,13^. We utilized this cut-off to test machine learning methods to predict this diagnostic threshold PaO_2_/FiO_2_ ≤ 150 for the different imputation techniques. We implemented three classification models including neural network, logistic regression, and a kernel-based model, SVM.

For each machine learning model, we applied a tenfold cross-validation and calculated the sensitivity, specificity, likelihood ratios, diagnostic Odds Ratio (OR), Area Under Receiver Operating Characteristic curve (AUROC), F1 score and Bayesian Information Criterion (BIC) to compare across models. The two neural network models for classification were similar to the neural networks used in regression, except the output layer used the sigmoid function. As with the regression models, various topologies were tested to arrive at the final two multi-layer perceptron (MLP) classifiers, one with an input size of seven features and the other with an input size of three features. The hidden layer size is (12, 8, 6, 4, 4) for the model with seven input features. For the other model which utilizes only three input features, we used two hidden layers of size 6 and 3. All hidden layers used the tangent activation function. We trained both models for 200 iterations with Adam optimizer, setting seven feature classifier momentum value as 0.8 and three feature classifier momentum value as 0.6. The learning rate was 0.001 and the batch size was 200 for both models.

In addition, we implemented a basic logistic regression model for classification purposes as well as the SVM model which classifies examples with an optimal hyperplane. For the logistic regression, it uses logistic function to model a binary dependent variable. We utilized the linear_model.LogisticRegression method provided in the scikit-learn library without regularization, and other arguments were set as default. For the SVM model, we compared the results by applying different kernels and the RBF kernel outperformed other kernels. Methods were similar to those used in the regression task.

### Comparison of machine-learning based algorithm to published non-linear and log-linear equations

We compared the performance of our machine learning algorithms to the previously published equations. For the non-linear equation from Brown et al.^11^ the PaO_2_ was imputed from the SpO_2_, where PO_2_ = PaO_2_, S = SpO_2_ and F = FiO_2_ which is illustrated in the Eq. (1). For situations where the recorded SpO_2_ was 100% (or, 1.0), the SpO_2_ was substituted with 0.996 given that the equation would not permit the calculation of S = 1.0.

Non-linear equation to impute PaO_2_ from the SpO_2_ (Reprinted with permission - see Acknowledgment section).

1 $$\begin{aligned} PO_{2} & = \left\{ {\frac{11,700}{{\left( {{\raise0.7ex\hbox{$1$} \!\mathord{\left/ {\vphantom {1 S}}\right.\kern-\nulldelimiterspace} \!\lower0.7ex\hbox{$S$}} - 1} \right)}} + \left[ {50^{3} + \left( {\frac{11,700}{{{\raise0.7ex\hbox{$1$} \!\mathord{\left/ {\vphantom {1 S}}\right.\kern-\nulldelimiterspace} \!\lower0.7ex\hbox{$S$}} - 1}}} \right)^{2} } \right]^{1/2} } \right\}^{1/3} \\ & \quad + \left\{ {\frac{11,700}{{\left( {{\raise0.7ex\hbox{$1$} \!\mathord{\left/ {\vphantom {1 S}}\right.\kern-\nulldelimiterspace} \!\lower0.7ex\hbox{$S$}} - 1} \right)}} - \left[ {50^{3} + \left( {\frac{11,700}{{{\raise0.7ex\hbox{$1$} \!\mathord{\left/ {\vphantom {1 S}}\right.\kern-\nulldelimiterspace} \!\lower0.7ex\hbox{$S$}} - 1}}} \right)^{2} } \right]^{1/2} } \right\}^{1/3} . \\ \end{aligned}$$

For the log-linear equation from Pandharipande et al.^11,13^, the PaO_2_/FiO_2_ was imputed from SpO_2_/FiO_2_ utilizing the Eq. (2):

Log-linear equation to impute PaO_2_ from the SpO_2_ (Reprinted with permission - see Acknowledgment section).

2 $$PO_{2} = F \cdot 10^{{\left( {0.48 + 0.78 \cdot log_{10} \left( \frac{S}{F} \right)} \right)}} .$$

### Sensitivity analysis

To compare the performance of our machine learning algorithms to previously published equations, a sensitivity analysis was performed by selecting either self-reported White or Black race. For each machine learning model, we implemented a tenfold cross-validation and calculated the sensitivity, specificity, likelihood ratios, diagnostic OR, AUROC, F1 score, RMSE (root-mean-square deviation), and BIC to compare across models.

## Results

### A parsimonious three features model is sufficient to impute PaO2/FiO_2_ ratio using a large dataset

**Figure 1:**
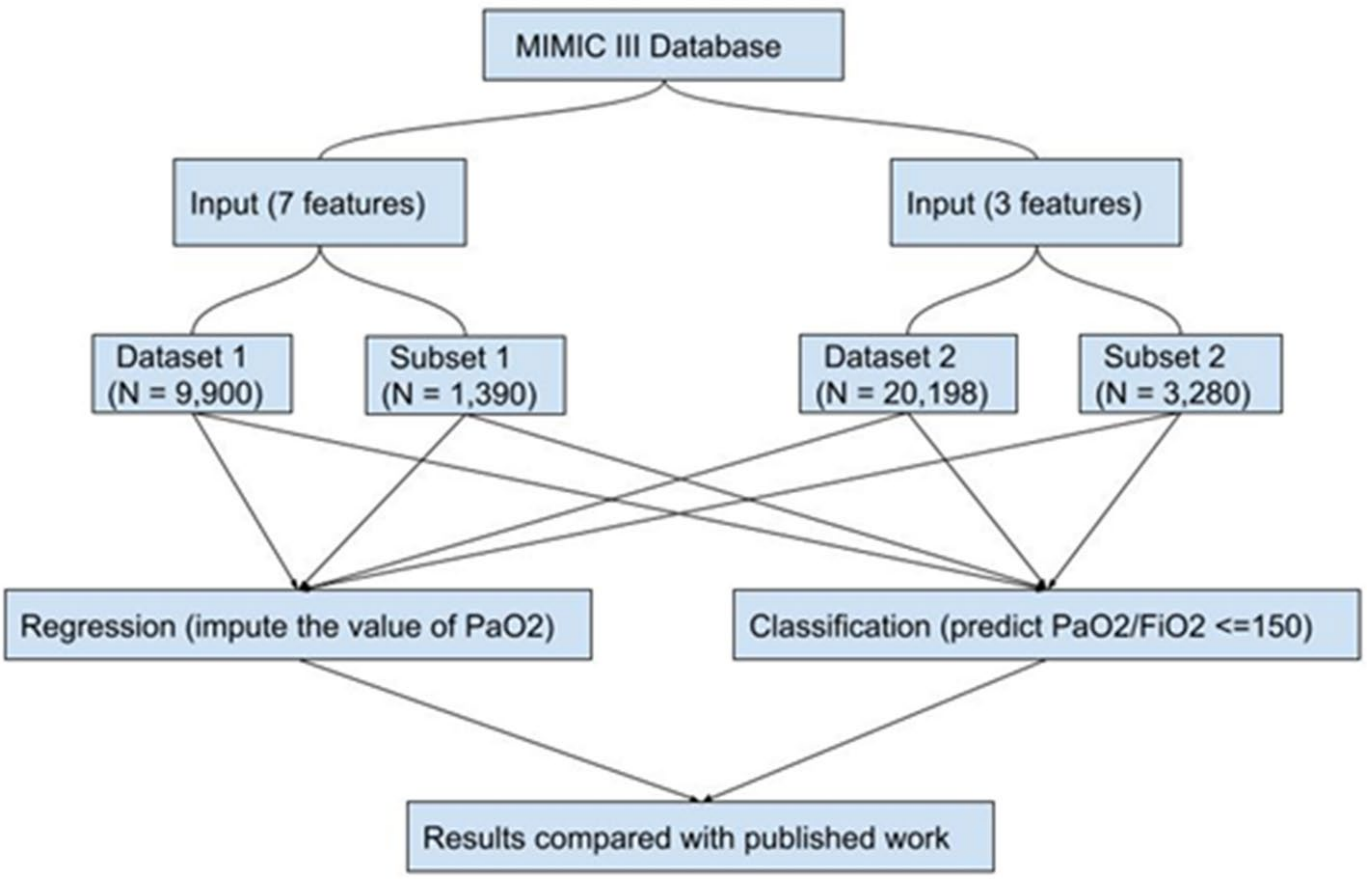
Overview of the experimental study design.

An overview of the machine learning tasks is outlined in Fig. 1. We initially chose seven relevant features from the chart events (SpO_2_, FiO_2_, TV, MAP, temperature, PEEP and vasopressor administration) representing recorded bedside measurements that were independent from an invasive arterial blood gas measurement. When applying the seven features to impute the PaO_2_, the final data set contained 9900 unique ICU encounters from 9302 mechanically ventilated patients (Supplementary Table e1). The relationship between SpO_2_/FiO_2_ (S/F) and the PaO_2_/FiO_2_ (P/F) was examined in dataset 1 containing 9900 unique ICU events from the MIMIC-III database and was best described by a log-linear relationship between the transformed logarithmic value of the SF and PF ratios as previously described by Pandharipande et al.^13^ (Supplementary Fig. e1). The relationship between S/F and P/F ratios showed high variance across the distribution of mechanically ventilated subjects (R^2^ = 0.21).

For the regression task, we derived the RMSE and BIC for each of the different seven feature machine learning models (neural network, linear regression, support vector regression) to assess the performance of the imputation techniques. The RMSE and BIC of the three machine learning methods are shown in Supplementary Table e2. All the machine learning models outperformed the previously published non-linear and log-linear equations as shown by lower RMSE score; the same was observed for subset 1 (SpO_2_ < 97%). For the classification task, the three machine learning methods achieved similar classification performance according to F1 scores, as shown in Supplementary Table e3; the same pattern was observed for subset 1 (SpO_2_ < 97%).

To improve practicality of the method at the bedside, we attempted to use the smallest number of features possible to predict the PaO_2_ or PaO_2_/FiO_2_ ratio from the regression and classification tasks, respectively. Compared to the other measured variables, PEEP had the strongest correlation with PaO_2_/FiO_2_ (r = − 0.31) outside of the SF ratio (SpO_2_/FiO_2_) (Table 1). Using this information, we created a 3-feature model using SpO_2_, FiO_2_ and PEEP. As compared to seven features, three features were sufficient to impute PaO_2_/FiO_2_ ratio with a similar degree of accuracy. The 3-feature model was therefore utilized in the remainder of the analysis for the machine learning algorithms. The final 3-feature data set (dataset 2) contained 20,198 ICU encounters from 17,818 unique patients (Table 2). Forty percent of subjects were of female sex and the mean age was 64 years. The degree of hypoxemic respiratory failure, as measured by the PaO_2_/FiO_2_ ratio^1^, showed a distribution in which 26% had mild respiratory failure (PaO_2_/FiO_2_ = 201–300), 22% had moderate respiratory failure (PaO_2_/FiO_2_ = 101–200), and 8% had severe respiratory failure (PaO_2_/FiO_2_ ≤ 100).

**Table 1:** Correlation coefficients between PF ratios and variables.

	SF ratio	PEEP	MAP	Temperature	Vasopressor administration	TV
PF ratio	0.44	− 0.31	0.06	− 0.06	− 0.04	0.02

Correlation coefficients between measured PF ratios and the six other measured variables (SpO_2_/FiO_2_ = SF ratio, PEEP, MAP, Temperature, Vasopressor Administration and TV) were performed. The variable with the strongest correlation coefficient (r) was chosen for the 3-feature model.

*PF ratio* PaO_2_/FiO_2_, *SF ratio* SpO_2_/FiO_2_, *TV* tidal volume, *PEEP* positive end-expiratory pressure, *MAP* mean arterial pressure.

**Table 2:** Subject characteristics based on three features.

**Total ICU events, N**	20,198
Female sex, n (%)	8084 (40.0)
Age in years, mean (± SD)^a^	64.0 (± 16.2)
PaO_2_/FiO_2_, mean (± SD)	310.4 (± 184.4)
**Available mean PaO**_**2**_**/FIO**_**2**_**, N**	20,198
PaO_2_/FiO_2_ > 300, n	8996
PaO_2_/FiO_2_ = 201–300, n	5226
PaO_2_/FiO_2_ = 101–200, n	4448
PaO_2_/FiO_2_ < 100, n	1528
**Available SpO**_**2**_** measurements per unique patient, N**	17,818
1 measurement, n	16,065
2 measurements, n	1367
3 measurements, n	262
4 measurements, n	77
5 measurements, n	29
6 measurements, n	14
7 measurements, n	4

The 3-feature models captured 20,198 ICU events from 17,818 unique patients. Variables included in the 3-feature machine learning models are SpO_2_, FiO_2_, and PEEP.

*ICU* intensive care unit.

^a^For subjects older than 89 years, the age was assigned as 90 years of age.

### Machine learning models show improved performance when compared to the prior published equations for regression

We quantitatively derived the RMSE for all of the machine learning and previously published models and the BIC for each of the three machine learning models to assess the performance of the different imputation techniques (Table 3). The RMSE of the neural network, linear regression and SVR machine learning models were 84.7, 88.8 and 85.9, respectively, compared to 117.7 and 91.8 for the log-linear and non-linear equations. The lower RMSE values indicate that the three machine learning models outperformed the previously published equations. Of the machine learning models, the neural network method showed the lowest RMSE as well as the lowest BIC in both the whole dataset (dataset 2) and for SpO_2_ < 97% (subset 2). A Bland–Altman Plot suggests that the neural network model is comparable to the published equations (Supplementary Fig. e2). There was decreasing accuracy at higher PaO_2_/FiO_2_ ratios for all the methods examined.

**Table 3:** RMSE and BIC of the 3-feature machine learning models regression tasks compared to published methods.

	Entire dataset 2 (20,198 events)		Subset 2 (SpO_2_ < 97%) (3280 events)	
	RMSE	BIC	RMSE	BIC
Neural network	84.7	17,952.7	67.5	2778.9
Linear regression	88.8	18,144.3	68.0	2783.5
Support vector regression	85.9	18,013.6	70.3	2805.0
Log-linear	117.7	NA	72.2	NA
Non-linear	91.8	NA	81.2	NA

The RMSE and BIC for the 3-feature machine learning models were calculated for the entire dataset (20,198 ICU events) and a subset of the dataset with SpO_2_ < 97% (3280 ICU events) and compared to the published log-linear and non-linear models.

*BIC* Bayesian information criterion, *RMSE* Root-mean-square deviation.

RMSE: An estimate of the differences between values predicted by a model and the values observed. The lower RMSE is, the lower the difference that exists between the predicted and observed values.

BIC: A criterion used in Bayesian statistics to choose between models. The model with the lowest BIC is supposed to be the best.

### Machine learning models show improved performance for the classification task

**Figure 2:**
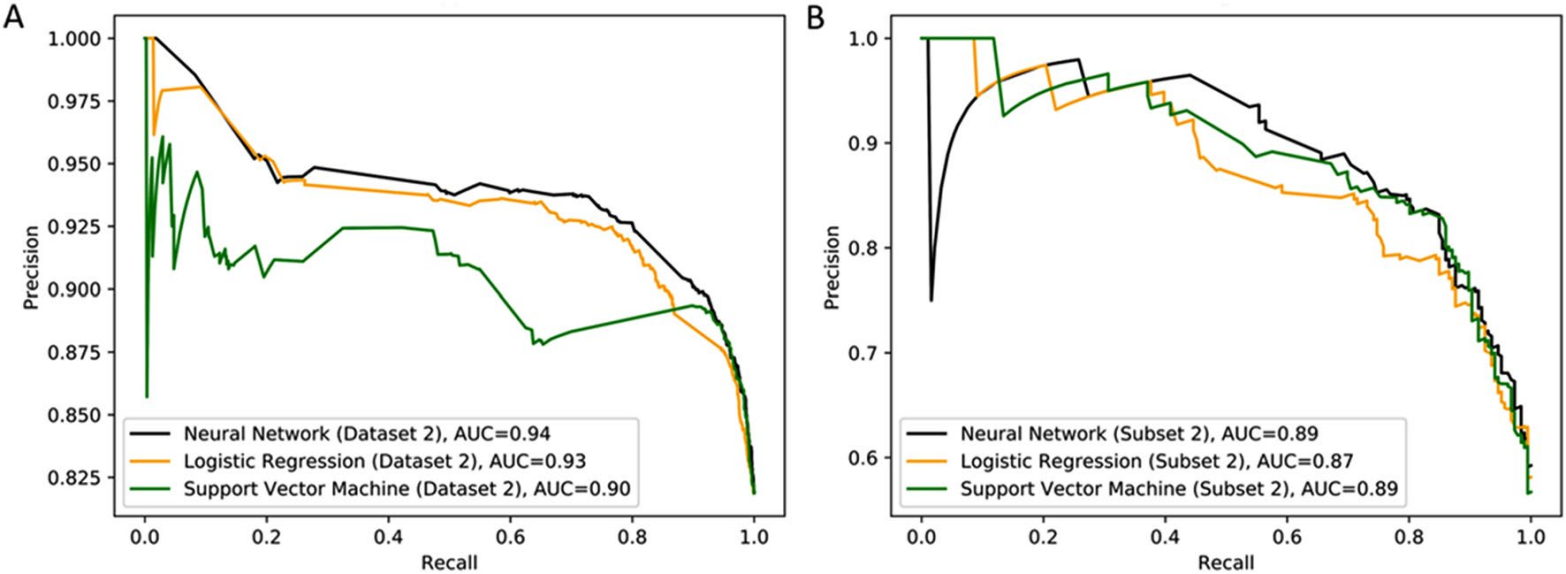
Precision-recall curves of machine learning models in Dataset 2 and Subset 2 using 3 features. The precision recall curves, where improved performance is demonstrated if the curve is closer to the upper right-hand corner or has the highest area under the curve (AUC), are shown for the 3 machine learning models for (**A**) the entire Dataset 2 (N = 20,198) ICU events) and (**B**) Subset 2 where SpO_2_ < 97% (N = 3280 ICU events). Data was obtained from the MIMIC-III database v1.4 (https://mimic.physionet.org).

We compared the performance of the machine learning models with the log-linear and non-linear equations using F1 scores. Similar to the findings for the regression task, all three machine learning models performed better in the whole dataset than log-linear and non-linear equations (Table 4). When the dataset was limited to SpO_2_ < 97% (subset 2), the machine-learning methods performed slightly better than log-linear and better than non-linear equations, respectively (Table 4). The F1 scores for all three machine learning methods were similar when using the whole dataset (dataset 2) and for subset 2 where SpO_2_ < 97%. As shown in Fig. 2, when comparing the 3 machine learning models to one another, the neural network preformed slightly better in the whole dataset (area under the precision recall curve = 0.94 for the neural network compared to 0.93 and 0.91 for the logistic regression and support vector machine model, respectively). The three models had similar performance in subset 2.

**Table 4:** Prediction performance of machine learning classification models based on three features.

	Entire dataset 2 (20,198 events)					Subset 2 (SpO_2_ < 97%) (3280 events)				
	Neural network	Logistic regression	SVM	Log-linear	Non-linear	Neural network	Logistic regression	SVM	Log-linear	Non-linear
Total, No	20,198	20,198	20,198	20,198	20,198	3280	3280	3280	3280	3280
Sensitivity	0.96	0.97	0.98	0.84	0.93	0.80	0.87	0.83	0.85	0.58
Specificity	0.39	0.26	0.33	0.56	0.49	0.76	0.59	0.69	0.59	0.89
Positive LR	1.59	1.32	1.46	1.90	1.83	3.37	2.13	2.75	2.09	5.16
Negative LR	0.09	0.10	0.07	0.29	0.15	0.27	0.23	0.25	0.25	0.47
Diagnostic OR	17.12	13.16	19.68	6.49	12.61	12.53	9.46	10.96	8.44	10.94
AUROC	0.83	0.81	0.74	NA	NA	0.85	0.83	0.84	NA	NA
F1	0.92	0.92	0.92	0.87	0.91	0.81	0.80	0.81	0.79	0.70
BIC	− 4612.6	− 4440.7	− 4446.0	NA	NA	− 591.8	− 567.0	− 580.0	NA	NA

Prediction performance statistics were calculated for the machine learning models based on three features and compared to the Log-linear and Non-linear methods for the entire dataset (20,198 ICU events; entire dataset 2) and for a subset of the events where SpO_2_ < 97% (3280 events; subset 2). Variables included in the 3-feature machine learning models are SpO_2_, FiO_2_, and PEEP.

*PaO*_*2*_*: SVR* Support vector regression, *AUROC* area under receiver operating characteristic curve, *BIC* Bayesian information criterion, *LR* likelihood ratio, *OR* odds ratio.

### Sensitivity analysis

Hidden hypoxemia, or the discrepancy between peripheral oxygen saturation (SpO_2_) measurements and the arterial oxygen saturation (SaO_2_) measured by ABG, was recently identified to occur in 5.3–5.5% of patients in the ICU setting^23,24^. Hidden hypoxemia, defined as SpO_2_ ≥ 88% despite an SaO_2_ ≤ 88%, was observed in all races and ethnic groups but occurs with higher prevalence in Black patients^23,24^. We conducted a sensitivity analysis to compare the performance of the machine learning models between self-reported Black and White race in dataset 2. For the regression task, among Black patients, machine learning algorithms outperformed both non-linear and log-linear equations in terms of the regression task (RMSE: 88.7, 91.1, 90.1, 117.4, and 95.8 for neural network, linear regression, SVR, log-linear, and non-linear models, respectively). Among machine learning algorithms, neural network revealed the highest performance in Black patients (Supplementary Table e4). Focusing on Black patients with SpO_2_ < 97% (subset 2), machine learning models showed superior performance over previously published equations (RMSE: 72.1, 74.4, 71.5, 85.0, and 95.6 for neural network, linear regression, SVR, log-linear, and non-linear models, respectively). The same pattern was observed for White patients in both the whole population and patients with SpO_2_ < 97% (subset 2) (RMSE in White patients: 84.6, 88.3, 85.9, 117.7, and 91.8; RMSE in White patients with SpO_2_ < 97%: 67.8, 68.3, 70.5, 72.2, and 81.2 for neural network, linear regression, SVR, log-linear, and non-linear models, respectively).

Considering the classification task, all machine learning algorithms performed better than or comparable to previously published equations in Black patients (F1: 0.93, 0.92, 0.93, 0.89, 0.92 for neural network, linear regression, SVR, log-linear, and non-linear models, respectively). Of note, neural network model performed slightly better than the other two machine learning algorithms in Black patients (AUC: 0.78, 0.77, 0.68 for neural network, logistic regression, and SVM model, respectively). Considering Black patients with SpO_2_ < 97% (subset 2), machine learning models outperformed conventional equations (F1: 0.82, 0.82, 0.84, 0.81, 0.73 for neural network, linear regression, SVR, log-linear, and non-linear models, respectively). Among White population, machine learning models outperformed conventional equations in both the whole population and patients with SpO_2_ < 97% (subset 2) (F1 in White patients: 0.92, 0.92, 0.92, 0.87, and 0.91; F1 in White patients with SpO_2_ < 97%: 0.81, 0.80, 0.81, 0.80, and 0.70 for neural network, linear regression, SVR, log-linear, and non-linear models, respectively), and neural network was the preferrable model. These findings are summarized in Supplementary Table e5.

### Machine learning algorithms show a better accuracy in the validation cohort

We developed an online calculator using the three machine learning algorithms requiring three inputs (SpO_2_, FiO_2_, and PEEP): https://dikb.org/pa02-predictor. The calculator was then utilized in an independent validation cohort of 133 mechanically ventilated ICU patients to impute the PaO_2_ in a regression task. The imputed PaO_2_ was compared to the actual PaO_2_ obtained by ABG. The accuracy of the machine learning algorithms was compared to the non-linear equation and was reported as the RMSE and adjusted R-squared (Table 5). The neural network and SMV models had lower RMSE than the previously published non-linear equation, demonstrating improved performance in the imputation of PaO_2_. Adjusted R-squared was also higher in the neural network and SMV models. To clarify the models proposed in this study, the following example is worth mentioning: with the assumption of SpO_2_ = 100%, FiO_2_ = 0.6, and PEEP = 5 cmH_2_O (observed PaO_2_/FiO_2_ = 190), the predicted PaO_2_ is estimated as 203.0, 186.2, 188.4 using neural network, SVM, and regression models, respectively, while the estimate of conventional non-linear model is 167 (Table 6).

**Table 5:** RMSE of the 3-feature machine learning models regression task compared to the published non-linear equation.

N = 133	Neural network	SVR	Regression	Non-linear
RMSE (adjusted R^2^)	65.0 (0.35)	64.9 (0.35)	74.1 (0.16)	67.1 (0.31)

The PaO_2_ was imputed using an online calculator of the three machine learning models using SpO_2_, PEEP, and FiO_2_ from a validation cohort of 133 mechanically ventilated ICU patients. Subsequently, the RMSE and adjusted R^2^ for the 3-feature machine learning models were calculated and compared to the published non-linear equation. A lower RMSE and higher adjusted R^2^ indicate higher accuracy.

*SVR* Support vector regression, *RMSE* root-mean-square deviation.

**Table 6:** Examples of comparing four models applied to four cases from different categories of PaO_2_ (< 150, 150–200, 200–300, > 300).

PaO_2_	SpO_2_	FiO_2_	PEEP	Neural network-imputed	Regression-imputed	SVR-imputed	Nonlinear-imputed
113	96	40	5	115.3	136.7	101.6	82
190	100	60	5	203.0	186.2	188.4	167
217	100	90	5	226.8	220.1	194.2	167
304	100	100	5	259.3	231.4	260.5	167

*PaO*_*2*_ Partial pressure of oxygen, *FiO*_*2*_ fraction of inspired oxygen, *SpO*_*2*_ peripheral saturation of oxygen, *PEEP* positive end-expiratory pressure, *SVR* support vector regression.

## Discussion

We used the publicly available MIMIC-III database as a derivation cohort to develop and evaluate machine-learning algorithms to impute PaO_2_ utilizing non-invasive SpO_2_ in patients who are mechanically ventilated. We tested three machine learning models (neural network, linear regression and SVR) first using seven available clinical variables SpO_2_, FiO_2_, PEEP, TV, MAP, temperature, and vasopressor administration to impute the PaO_2._ We subsequently used a parsimonious model with three clinical variables (SpO_2_, FiO_2_ and PEEP) to non-invasively impute PaO_2_ in both a derivation and validation cohort. The imputation of PaO_2_ from the regression tasks enabled us to derive the PaO_2_/FiO_2_, a clinically meaningful ratio with predictive value^1,25^. Additionally, we performed a classification task to predict PaO_2_/FiO_2_ ≤ 150, a cut off that has been used to capture those patients with moderate to severe respiratory failure in ARDS cohorts^11,13^ and to guide patient management^5^. To increase the clinical applicability of our work, we also developed an open-access online calculator to impute the PaO_2_ using the 3-feature model requiring only non-invasive bedside parameters in mechanically ventilated patients. Our calculator showed improved accuracy in the imputation of the PaO_2_ when compared to the previously published non-linear equation in both our initial cohort and the validation cohort.

To develop the machine learning algorithms, we initially evaluated clinical variables such as PEEP, TV, MAP, temperature, and vasopressor administration that are easily obtained at the bedside. TV, MAP, temperature and vasopressor use demonstrated a stochastic distribution and did not significantly alter the accuracy of the machine-learning based algorithms and were therefore removed to create the 3 features model (SpO_2_, FiO_2_, PEEP). This 3-feature model provides a framework for generalizability using large datasets of mechanically ventilated patients.

We considered other clinical variables such as skin pigmentation, pulse oximeter location, oximeter manufacturer, vasopressor infusion, and laboratory variables such as serum bicarbonate, serum chloride, serum creatinine, serum sodium but others have shown these variables provided negligible improvement in the accuracy of imputation in a prior prospective study^11^ and were therefore not included. However, it is worth mentioning that recent studies showed discrepancy between peripheral oxygen saturation (SpO_2_) measurements and the arterial oxygen saturation (SaO_2_) measured by ABG. This discrepancy, defined as SpO_2_ ≥ 88% despite an SaO_2_ ≤ 88% and referred to as hidden hypoxemia, was present in all racial and ethnic groups but showed higher prevalence in Black patients^23,24^. Considering this discrepancy between SpO_2_ and arterial oxygen saturation occurs more frequently in Black patients^24^, we performed a sensitivity analysis showing that our machine learning algorithms outperform previously published equations both in the Black and White race.

Our study shows that a machine learning based method for both the regression and classification task, when applied to the MIMIC-III critical care database, improved the accuracy compared to the previously published non-linear and log-linear imputation methods. As is evidenced by comparing the F1 and discrimination measures in Table 4, the performance improvement was more modest for the classification task in subset 2 where SpO_2_ < 97%. A possible explanation is that there were fewer ICU events (smaller N) per group in the subset.

Prior studies have examined the relationship between SF and PF ratios for patients with ARDS to determine whether the non-invasive SF ratio can be substituted for the invasively obtained PF ratio^11,13,26^. Panharipande, et al. studied matched measurements of SpO_2_ and PaO_2_ in a heterogeneous population (i.e., patients undergoing general anesthesia and patients with ARDS) to determine the association between SF and PF ratios in order to calculate the respiratory parameter of the SOFA score^13^. In their study, matched SpO_2_ and PaO_2_ values were obtained from two groups of patients: Group 1 comprised of the derivation set and was obtained from patients undergoing general anesthesia from a single center and Group 2 comprised a validation set utilizing data from patients enrolled in a multi-center randomized clinical trial examining low versus high tidal volume for acute respiratory management of ARDS (ARMA)^27^. All SpO_2_ values > 97% were also excluded from analysis in order to maximize matched data to those values likely to be within the linear range of the oxyhemoglobin dissociation curve. Data from 4728 matched SpO_2_ and PaO_2_ measurements showed that the relationship was best described by a log-linear equation with slight variation based upon the level of PEEP. In the setting of a more heterogeneous population, a poorer correlation was noted between SF and PF ratios. The regression equation of Log(PF) = 0.48 + 0.78 × Log(SF) yielded an R-square of 0.31^13^.

Additionally, a retrospective analysis of arterial blood gas measurements from three ARDS Network studies compared the performance of non-linear, log-linear and linear imputation methods to derive PaO_2_ from the SpO_2_^12^. In all patients (N = 1184), the nonlinear imputation was equivalent to log-linear imputation. However, in those patients with SpO_2_ < 97% (N = 707), the nonlinear imputation showed lower error than either linear or log-linear equations. A prospective study was subsequently conducted in patients enrolled in the Prevention and Early Treatment of Acute Lung Injury network^11^ to assess the performance of the non-linear equation to impute PaO_2_ from the SpO_2_ and compare it to the prior log-linear and linear equations^11,13,26^. This study included 1034 arterial blood gases from 703 patients, of which 650 arterial blood gases had matched SpO_2_ < 97%. The non-linear equation showed lower error and better identified moderate to severe ARDS patients (defined in the study as PaO_2_/FiO_2_ ≤ 150) when compared to log-linear or linear imputation methods.

In our study, we similarly found a high degree of variance across SpO_2_ values and corresponding measured PaO_2_ values which was noted when we formally examined the relationship between SF and PF. This may be attributed to the retrospective nature of the data collection and the numerous variables that may confound the reliability of a recorded SpO_2_ measured non-invasively to reflect the arterial SaO_2_^8,10,12^. Despite this limitation, the machine learning algorithms performed better on both regression and classification tasks when compared to the log-linear and non-linear published equations.

We used a validation cohort to show improved accuracy for the neural network and kernel-based machine learning algorithms when compared to the previously published non-linear equation. Another strength of our study is the development of an online calculator that can be used to impute the PaO_2_ from three noninvasive parameters (SpO_2_, FiO_2_ and PEEP) and may serve as a tool for future studies in large electronic health record datasets. Additionally, our machine learning models allow for the evaluation of all mechanically ventilated patients with available data rather than narrowing the analysis to a specific population such as those with ARDS. Given the inclusion of all mechanically ventilated patients, a significant number of SpO_2_ values were > 97% (N = 8510 for seven features and N = 16,918 for three features). While this reduced the accuracy of the imputed PF ratio, particularly above a certain threshold, the machine learning models were applied to the data without a pre-defined restriction placed upon the range of SpO_2_ values and showed better performance than both the log-linear and non-linear equations on both the regression and classification tasks.

Imputation of PaO_2_ from SpO_2_ has been increasingly implemented in clinical and research settings using previously published equations for subjects that do not have invasive ABG measurements readily available. This underscores the need to improve upon existing published equations and the clinical importance of machine learning models proposed. Machine learning models are currently being used to answer numerous clinical questions; these models have substantially impacted different scopes of medicine from early-warning systems for sepsis to imaging diagnostics^24^. Herein, we proposed three machine learning algorithms which can provide a framework for future investigations. The online calculator, on the other hand, can provide feasible prediction of PF ratio from SF ratio at the bedside for clinicians working in the critical care settings.

We showed that machine learning models outperformed previously published equations in terms of imputing PaO_2_ from SpO_2_ in the mechanically-ventilated adult population. Consistent with our findings, Sauthier et al., utilized neural network models to validate a continuous and noninvasive method of hypoxemia estimation in pediatric population^28^. They utilized convolutional neural network (CNN), long short-term memory network (LSTM), and multilayer perceptron (MLP) to impute PaO_2_. Intriguingly, they concluded that bias was lowered when using neural network models compared to mathematical equations.

In summary, any of the tested machine learning models applied to MIMIC-III dataset enabled imputation of PaO_2_ from the SpO_2_ with lower error and provided greater accuracy in predicting PaO_2_/FiO_2_ ≤ 150 across the entire range of SpO_2_ examined when compared to that of published equations in two independent cohorts. All machine learning models proposed in this paper outperformed log-linear and non-linear equations. Future work will be required to prospectively test ML algorithms for use in clinical practice. Additionally, our study provides a clinically relevant online calculator for the imputation of the PaO_2_ from the 3-feature machine learning models. The calculator requires the input of SpO_2_, FiO_2_, and PEEP all of which are non-invasive and readily available at the bedside of mechanically ventilated patients.

## Supplementary Information

Supplementary Information.

## Abbreviations

PaO_2_: Partial pressure of oxygen
FiO_2_: Fraction of inspired oxygen
SpO_2_: Peripheral saturation of oxygen
PF ratio: PaO_2_/FiO_2_
SF ratio: SpO_2_/FiO_2_
ARDS: Acute respiratory distress syndrome
SOFA: Sequential organ failure assessment
ABG: Arterial blood gas
TV: Tidal volume
PEEP: Positive end-expiratory pressure
MAP: Mean arterial pressure
SVR: Support vector regression
RBF: Radical basis function kernel
AUROC: Area under receiver operating characteristic curve
BIC: Bayesian information criterion
RMSE: Root-mean-square deviation
