# Supplementary material for: Machine learning based algorithms to impute PaO2 from SpO2 values and development of an online calculator
Source: Sci Rep. 2022 May 17;12:8235. doi: 10.1038/s41598-022-12419-7 (PMC9114384; doi:10.1038/s41598-022-12419-7)
Supplement: Supplementary file 1 — Supplementary Information. [file 41598_2022_12419_MOESM1_ESM.docx]

**Supplementary Materials:**

**Supplementary Tables:**

| Total ICU events, N | 9,900 |
| --- | --- |
| Female sex, n (%) | 3,713 (37.5) |
| Age in years, mean (+ SD)^*^ | 64.0 (+ 16) |
| PaO2/FIO2, mean (+ SD) | 322.8 (+193.0) |
|  |  |
| Available mean PaO2/FIO2, N | 9,900 |
| PaO2/FIO2 > 300, n | 4,656 |
| PaO2/FIO2 = 201-300, n | 2,468 |
| PaO2/FIO2 = 101-200, n | 2,042 |
| PaO2/FIO2 < 100, n | 734 |
|  |  |
| Available SpO2 measurements per unique patient, N | 9,302 |
| 1 measurement, n | 8797 |
| 2 measurements, n | 433 |
| 3 measurements, n | 58 |
| 4 measurements, n | 9 |
| 5 measurements, n | 4 |
| 6 measurements, n | 0 |
| 7 measurements, n | 1 |

**Supplementary Table e1. Subject Characteristics in the 7 features models**. The 7 feature models captured 9,990 ICU events from 9,302 unique patients. Variables included in the 7 features machine learning models are SpO2, FiO2, TV, MAP, temperature, PEEP and vasopressor administration. ^*^For subjects older than 89 years, the age was assigned as 90 years of age.

|  | **Entire Dataset 1**  **(9,900 events)** | | **Subset 1 (SpO2 < 97%)**  **(1,390 events)** | |
| --- | --- | --- | --- | --- |
|  | **RMSE** | **BIC** | **RMSE** | **BIC** |
| **Neural Network** | **86.2** | **8873.1** | **66.6** | **1196.8** |
| **Linear Regression** | 92.1 | 9002.9 | 67.1 | 1199.0 |
| **Support Vector Regression** | 87.5 | 8902.3 | 68.5 | 1203.5 |
| **Log-linear** | 130.2 | NA | 73.1 | NA |
| **Non-linear** | 97.7 | NA | 82.4 | NA |

**Supplementary Table e2. RMSE and BIC of the 7 features machine learning models compared to published methods**. The RMSE and BIC for the 7 features models were calculated for the entire dataset (9,900 ICU events) and a subset of the dataset with SpO2 < 97% (1,390 ICU events) and compared to the published log-linear and non-linear models.

|  | **Entire Dataset 1**  **(9,900 events)** | | | | | **Subset 1 (SpO2 < 97%)**  **(1,390 events)** | | | | |
| --- | --- | --- | --- | --- | --- | --- | --- | --- | --- | --- |
|  | **Neural**  **Network** | **Logistic Regression** | **SVM** | **Log-linear** | **Non-linear** | **Neural Network** | **Logistic regression** | **SVM** | **Log-linear** | **Non-linear** |
| **Total, No.** | 9,900 | 9,900 | 9,900 | 9,900 | 9,900 | 1,390 | 1,390 | 1,390 | 1,390 | 1,390 |
| **Sensitivity** | 0.96 | 0.98 | 0.98 | 0.80 | 0.93 | 0.79 | 0.84 | 0.81 | 0.82 | 0.53 |
| **Specificity** | 0.43 | 0.28 | 0.33 | 0.62 | 0.52 | 0.77 | 0.68 | 0.71 | 0.65 | 0.92 |
| **Positive**  **LR** | 1.71 | 1.37 | 1.48 | 2.12 | 1.94 | 3.73 | 2.68 | 2.91 | 2.36 | 6.37 |
| **Negative**  **LR** | 0.09 | 0.07 | 0.06 | 0.33 | 0.13 | 0.28 | 0.24 | 0.27 | 0.27 | 0.51 |
| **Diagnostic**  **OR** | 20.08 | 18.96 | **25.48** | 6.43 | 15.04 | **13.45** | 11.42 | 10.83 | 8.67 | 12.40 |
| **AUROC** | **0.84** | 0.82 | 0.79 | NA | NA | 0.85 | 0.84 | **0.85** | NA | NA |
| **F1** | **0.93** | 0.93 | **0.93** | 0.85 | 0.92 | **0.79** | **0.79** | 0.79 | 0.78 | 0.66 |
| **BIC** | **-2312.40** | -2212.10 | -2243.50 | NA | NA | -221.30 | -216.40 | **-222.30** | NA | NA |

**Supplementary Table e3. Prediction performance of machine learning classification models based on 7 features.** Prediction performance statistics were calculated for the machine learning models based on 7 features and compared to the Log-linear and Non-linear methods for the entire dataset (9,900 ICU events) and for a subset of the events where SpO2 <97% (1,390 ICU events). Variables included in the 7 features machine learning models are SpO2, FiO2, TV, MAP, temperature, PEEP and vasopressor administration.

| **Self-reported Black Race** | | | | |
| --- | --- | --- | --- | --- |
|  | **Dataset 2**  **(1,421 events)** | | **Subset 2 (SpO2 < 97%)**  **(216 events)** | |
|  | **RMSE** | **BIC** | **RMSE** | **BIC** |
| **Neural Network** | 88.7 | 1290.5 | 72.1 | 191.8 |
| **Linear Regression** | 91.1 | 1298.2 | 74.4 | 193.4 |
| **Support Vector Regression** | 90.1 | 1294.6 | 71.5 | 188.0 |
| **Log-linear** | 117.4 | NA | 85.0 | NA |
| **Non-linear** | 95.8 | NA | 95.6 | NA |
| **Self-reported White Race** | | | | |
|  | **Dataset 2**  **(14,386 events)** | | **Subset 2 (SpO2 < 97%)**  **(2,328 events)** | |
|  | **RMSE** | **BIC** | **RMSE** | **BIC** |
| **Neural Network** | 84.6 | 12790.3 | 67.8 | 1978.6 |
| **Linear Regression** | 88.3 | 12911.4 | 68.3 | 1982.4 |
| **Support Vector Regression** | 85.9 | 12832.5 | 70.5 | 1996.9 |
| **Log-linear** | 117.7 | NA | 72.2 | NA |
| **Non-linear** | 91.8 | NA | 81.2 | NA |

**Supplementary Table e4. RMSE and BIC of the 3-features machine learning models compared to published methods based on self-reported Black and White race**. The RMSE and BIC for the 3 features models were calculated for the subpopulation of Black and White race (1421 and 14386 ICU events, respectively) and a subset of the dataset with SpO2 < 97% (216 and 2328 ICU events, respectively) and compared to the published log-linear and non-linear models.

| **Self-reported Black Race** | | | | | | | | | | |
| --- | --- | --- | --- | --- | --- | --- | --- | --- | --- | --- |
|  | **Dataset 2**  **(1,421 events)** | | | | | **Subset 2 (SpO2 < 97%)**  **(216 events)** | | | | |
|  | **Neural**  **Network** | **Logistic Regression** | **SVM** | **Log-linear** | **Non-linear** | **Neural Network** | **Logistic regression** | **SVM** | **Log-linear** | **Non-linear** |
| **Sensitivity** | 0.98 | 0.98 | 0.99 | 0.88 | 0.94 | 0.89 | 0.88 | 0.92 | 0.83 | 0.61 |
| **Specificity** | 0.23 | 0.19 | 0.18 | 0.48 | 0.41 | 0.52 | 0.50 | 0.52 | 0.58 | 0.90 |
| **Positive**  **LR** | 1.28 | 1.21 | 1.21 | 1.70 | 1.58 | 1.95 | 1.85 | 1.95 | 2.00 | 5.89 |
| **Negative**  **LR** | 0.08 | 0.11 | 0.05 | 0.26 | 0.15 | 0.26 | 0.26 | 0.16 | 0.28 | 0.43 |
| **Diagnostic**  **OR** | 16.0 | 11.0 | 24.2 | 6.5 | 10.5 | 7.5 | 7.1 | 12.2 | 7.1 | 13.7 |
| **AUROC** | 0.78 | 0.77 | 0.68 | NA | NA | 0.80 | 0.81 | 0.80 | NA | NA |
| **F1** | 0.93 | 0.92 | 0.93 | 0.89 | 0.92 | 0.82 | 0.82 | 0.84 | 0.81 | 0.73 |
| **BIC** | -1590.63 | -1563.94 | -1534.64 | NA | NA | -171.33 | -175.49 | -173.57 | NA | NA |
| **Self-reported White Race** | | | | | | | | | | |
|  | **Dataset 2**  **(14,386 events)** | | | | | **Subset 2 (SpO2 < 97%)**  **(2,328 events)** | | | | |
|  | **Neural**  **Network** | **Logistic Regression** | **SVM** | **Log-linear** | **Non-linear** | **Neural Network** | **Logistic regression** | **SVM** | **Log-linear** | **Non-linear** |
| **Sensitivity** | 0.96 | 0.98 | 0.98 | 0.83 | 0.93 | 0.82 | 0.88 | 0.83 | 0.86 | 0.59 |
| **Specificity** | 0.39 | 0.25 | 0.31 | 0.56 | 0.48 | 0.70 | 0.57 | 0.69 | 0.59 | 0.88 |
| **Positive**  **LR** | 1.58 | 1.30 | 1.41 | 1.91 | 1.80 | 2.78 | 2.06 | 2.71 | 2.09 | 4.95 |
| **Negative**  **LR** | 0.10 | 0.10 | 0.08 | 0.29 | 0.15 | 0.25 | 0.22 | 0.25 | 0.24 | 0.47 |
| **Diagnostic**  **OR** | 15.8 | 13.0 | 17.6 | 6.6 | 12.0 | 11.1 | 9.4 | 10.8 | 8.7 | 10.5 |
| **AUROC** | 0.82 | 0.81 | 0.75 | NA | NA | 0.84 | 0.83 | 0.83 | NA | NA |
| **F1** | 0.92 | 0.92 | 0.92 | 0.87 | 0.91 | 0.81 | 0.80 | 0.81 | 0.80 | 0.70 |
| **BIC** | -16477.87 | -15937.42 | -15819.08 | NA | NA | -2116.91 | -2046.82 | -2087.63 | NA | NA |

**Supplementary Table e5. Prediction performance of machine learning classification models based on 3 features.** Prediction performance statistics were calculated for the machine learning models based on 3 features in the subpopulation of Black and White race and compared to the Log-linear and Non-linear methods for the related dataset (1421 and 14386 ICU events, respectively) and for a subset of the events where SpO2 <97% (216 and 2328 ICU events, respectively).


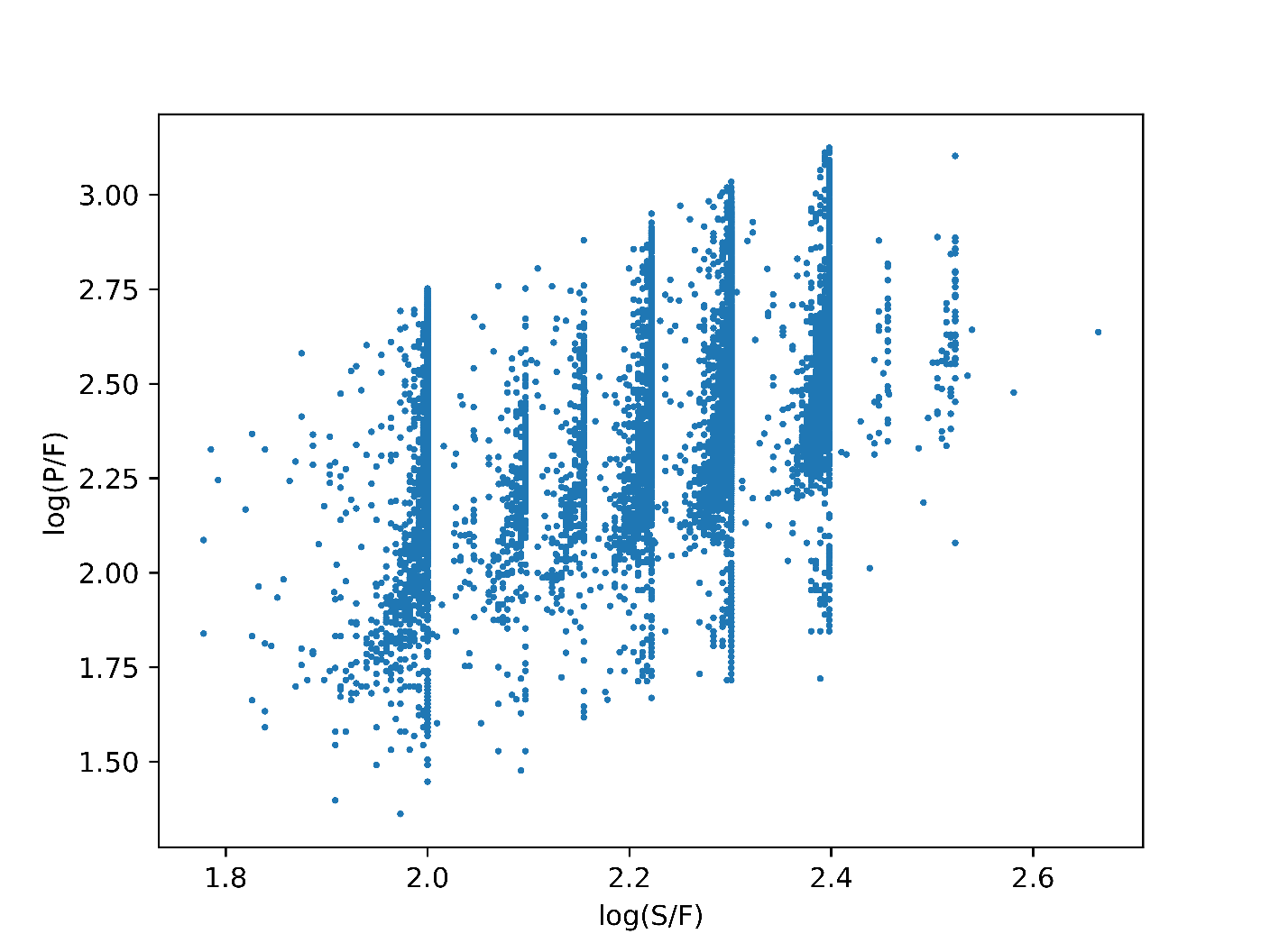


**Supplementary Figure e1. Log linear regression between SpO2/FIO2 (S/F) and PaO2/FIO2 (P/F).** The log-linear relationship between the transformed logarithmic value of the SF and PF ratios is shown. Dataset 1 includes 9,900 ICU events. R^2^ = 0.21. Each point represents a unique ICU event.


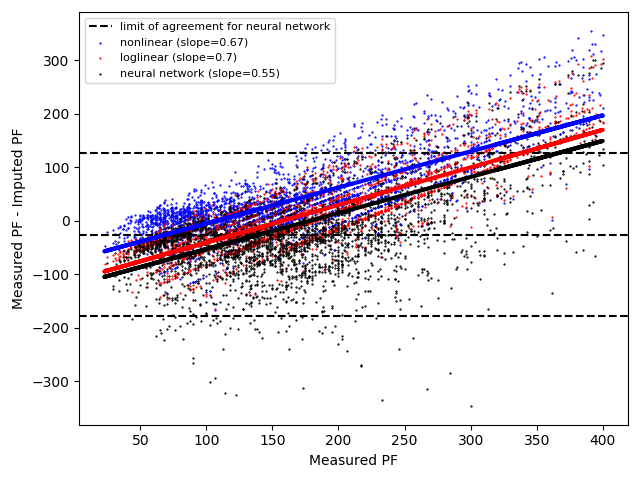


**Supplementary Figure e2: Bland-Altman Plots of imputed versus measured PaO2/FIO2 ratios comparing the published equations and the 3 features Neural Network machine-learning algorithm for Subset 2 (SpO2 < 97%).** Bland-Altman plots of imputed versus measured PaO2/FiO2 ratio comparing the Neural Network machine learning algorithm using 3 features to the published log-linear and non-linear equations. Subset 2 of the entire dataset 2 where SpO2 <97% (3,280 ICU events) is shown and the slope of each least squared line is reported. Each point represents a single ICU event.
